# A Review of the Most Frequent Compounds, Metals, and Compound and Metal Mixtures Found at U.S. Superfund Sites and Their Carcinogenic Potential

**DOI:** 10.1021/acs.chemrestox.4c00506

**Published:** 2025-06-03

**Authors:** June K. Dunnick, Charles P. Schmitt, Darlene Dixon

**Affiliations:** †Systems Toxicology Branch, ‡Office of Data Science, and §Mechanistic Toxicology Branch, Division of Translational Toxicology, National Institute of Environmental Health Sciences, National Institutes of Health, Research Triangle Park, North Carolina 27709, United States

## Abstract

The United States Environmental Protection Agency’s (U.S. EPA) National Priorities List (NPL) is a list of sites in the U.S. and its territories of national priority that are sources of known hazardous contaminants, pollutants, or substances that pose a significant risk to human health and the environment. These sites are commonly termed U.S. Superfund sites and contain many harmful compounds and metals. This paper reviews the carcinogenic potential of the most frequent compounds, metals, and mixtures at U.S. Superfund sites. Of the most frequent compounds and metals identified at U.S. Superfund sites, some are classified as human carcinogens and some as probable/possible human carcinogens. The most frequent mixtures of three individual carcinogenic compound or metals at U.S. Superfund sites include: nickel, arsenic, and cadmium (496 sites); benzene, arsenic, trichloroethene (451 sites); benzene, vinyl chloride, trichloroethene (420 sites); and arsenic, vinyl chloride, trichloroethene (386 sites). Many compounds or metals that are frequently found at U.S. Superfund Sites have not been evaluated for carcinogenic activity because of limited data including copper, xylene, mercury, barium, and iron. Factors in human cancer development include both environmental factors and genetic disease susceptibility backgrounds. Thus, future mixture toxicology studies should be conducted with a design that looks at mixture toxicology in a variety of models with varied genetic backgrounds.

## Graphical abstract

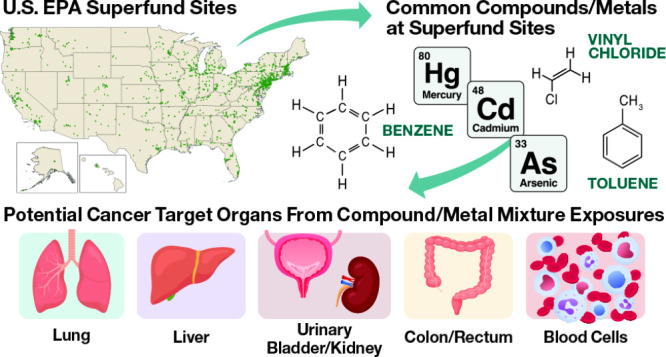

## Introduction

Environmental exposures to compounds and metals can contribute to the development of cancer,^1–3^ a leading cause of death in the U.S.^4^ Communities living near U.S. Superfund sites have the potential to be exposed to carcinogenic compounds and metals found at these sites.^5^ This paper reviews the carcinogenic potential of the compounds/metals and mixtures most frequently found at U.S. Superfund sites as reported by the National Toxicology Program (NTP) 2-year rodent bioassays technical documents,^6^ the NTP Report on Carcinogens (RoC),^7^ and the International Agency for Research on Cancer (IARC).^2^

The United States Environmental Protection Agency (U.S. EPA) National Priorities List (NPL) is a list of sites in the U.S. and its territories of national priority that contain known hazardous contaminants, pollutants, or substances that are deemed to pose significant risk to human health and the environment. There is a continuing effort to remove hazardous compounds, metals, and mixtures from many of the over 1000 federally recognized contaminated sites in the U.S. and its territories, but it may take decades to complete the cleanup.^8^ In 1980, the U.S. Congress passed the “Comprehensive Environmental Response, Compensation, and Liability Act (CERCLA)”,^9^ which included legislation for “long-term remedial response actions, to permanently and significantly reduce the dangers associated with releases or threats of releases of hazardous substances that are serious, but not immediately life threatening”. This act was reauthorized in 1986 and required the U.S. EPA to ensure that it assessed the relative degree of risk to human health and the environment posed by uncontrolled hazardous waste sites that may be placed on the NPL.^8^ The toxicity of not only a single compound exposure at these waste sites but also exposure to compound/metal mixtures at these sites is of concern.

Compounding the concern for exposure to toxic and carcinogenic compounds/metals/mixtures at U.S. Superfund sites are other social factors and noncompound stressors including decreased access to health care, food insecurity, and unfavorable housing conditions that may add to disease susceptibility.^10^ Prior to a 1994 Executive Order,^11^ there were biases in the cleanup of U.S. Superfund sites located in neighborhoods where vulnerable populations lived but support of cleaning up sites located in areas with an educated population.^12^

Traditional toxicology and cancer studies are usually conducted on one compound or metal at a time,^13^ and subsequent human health evaluations are also often conducted on one compound or metal at a time.^6^ Understanding cancer hazards from mixture exposures will allow a more complete evaluation of the human health hazards from environmental exposures. Studies have shown that ∼50% of human cancers are considered due to environmental factors and ∼50%, due to genetic background.^14^ Additional studies on the cancer potential of compound and metal mixtures frequently found at U.S. Superfund sites will help in our understanding of the potential health hazards from environmental exposures.^15^

## Methodology

### Compounds and Metals Identified at U.S. Superfund Sites

Using the U.S. EPA U.S. Superfund List 10,^16^ the most frequently found compounds, metals, and compound and metal mixtures at U.S. Superfund sites were analyzed to determine the cancer hazard potential of single compounds or metals and combinations (mixtures) of these compounds and metals.

### Evaluation of the Cancer Potential of Compounds and Metals Found at Superfund Sites Using National Toxicology Program Data

The carcinogenic potential of compounds and compound/metal mixtures was evaluated using data from the National Toxicology Program (NTP) Report on Carcinogens (RoC).^7,17^ *Salmonella* results were from NTP genetic toxicity tests.^18^

The NTP RoC, which was used as a primary source for the cancer potential of the top 30 most frequently found compounds and metals uses the following cancer classification scheme^7,17^ to categorize human carcinogens: “Known To Be Human Carcinogen” – there is sufficient evidence of carcinogenicity from studies in humans, which indicates a causal relationship between exposure to the agent, substance, or mixture, and human cancer; “Reasonably Anticipated To Be Human Carcinogen (RAHC)” – there is limited evidence of carcinogenicity from studies in humans, but there is sufficient evidence for cancer in animal model studies.

Additional information provided for the top 30 most frequently found compounds and metals at U.S. Superfund sites was from the NTP cancer rodent studies.^6^ The NTP rodent study conclusions included “Clear evidence of carcinogenic activity” demonstrated by studies that are interpreted as showing a dose-related (i) increase of malignant neoplasms, (ii) increase of a combination of malignant and benign neoplasms, or (iii) marked increase of benign neoplasms if there is an indication from this or other studies showing the ability of such tumors to progress to malignancy; “Some evidence of carcinogenic activity” demonstrated by studies that are interpreted as showing a compound-related increased incidence of neoplasms (malignant, benign, or combined) in which the strength of the response is less than that required for clear evidence; “Equivocal evidence of carcinogenic activity” demonstrated by studies that are interpreted as showing a marginal increase of neoplasms that may be compound related; and “No evidence of carcinogenic activity” demonstrated by studies that are interpreted as showing no compound-related increases in malignant or benign neoplasms. Inadequate study of carcinogenic activity is demonstrated by studies that, because of major qualitative or quantitative limitations, cannot be interpreted as valid for showing either the presence or the absence of carcinogenic activity. NTP *Salmonella* toxicity test results are added to help understand cancer mechanisms.^19^

### Evaluation of Cancer Potential Using Other Data Sets

The International Agency for Research on Cancer (IARC)^2^ cancer classifications for the most frequent U.S. Superfund compounds/metals are also provided for the top 30 most frequently found compounds and metals as additional information to the RoC cancer classifications.

The IARC Classification for carcinogens^2^ involves classification into Groups 1–3: “Group 1” – The agent (mixture) is carcinogenic to humans. The exposure circumstance entails exposures that are carcinogenic to humans; “Group 2A” – The agent (mixture) is probably carcinogenic to humans. The exposure circumstance entails exposures that are probably carcinogenic to humans; “Group 2B” (the agent (mixture) is possibly carcinogenic to humans. The exposure circumstance entails exposures that are possibly carcinogenic to humans; and “Group 3” – The agent (mixture or exposure circumstance) is not classifiable as to its carcinogenicity to humans.

### Data Processing

The data for this project were generated through a data processing pipeline consisting of the following stages:

(1) Downloaded data sets of compounds and metals at U.S. Superfund sites.^16^
(2) Prepared data sets (removed missing rows, relabeled columns to support merging of data sets).
(3) Reorganized data sets to a format with one compound and multiple findings per row. This step was necessary as reporting agencies often report findings for multiple compounds on the same row or multiple findings are reported for a compound on separate rows (typically when a compound was reported on in separate studies).
(4) Combined sets of similar compounds (e.g., different nickel or chromium compounds) as one set of compounds found at the U.S. Superfund sites. The compounds (e.g., nickel compounds) are treated as individual compounds in the subsequent processing steps and the individual compounds that constitute the compounds are removed. This was done for chromium­(VI) compound (18540-29-9, 7789-12-0, 7778-50-9, 13007-92-6, 7775-11-3, 7778-50-9); nickel compound (1313-99-1, 12035-72-2, 7440-02-0); chromium­(III) compound (16065-83-1, 27882-76-4, 14639-25-9); and arsenic compound (7778-39-4, 1327-53-3, 1303-00-0, 64436-13-1, 7440-38-2).
(5) Merged data sets into a combined file for analysis, dropping all compounds that do not appear in the EPA Superfund site list.^16^
(6) Computed a single compound hazard score for each compound.
(7) Computed a mixture compound hazard score for each mixture.

CAS registry numbers were used to identify equivalent compounds across the data sets. Automated software and manual quality-assessment checks were conducted to ensure that each pipeline stage performed as described in this section, and a final set of software tests compared the hazard scores from stages 6 and 7 versus hand-computed scores for each possible set of conditions.

## Results

### Overview of Compounds, Metals, and Mixtures Identified at the U.S. Superfund Sites

The most frequent organic compounds and metals identified in 1582 Superfund sites are found at sites across the country. There were a total of 1042 different individual compounds/metals present in U.S. Superfund sites based on unique names with 761 unique CAS registry numbers (Supplement One). Seven hundred of the unique CAS registry numbers are valid CAS registry numbers (using the Python casregnum package). Individual CAS registry numbers may not be found for some compound classes (e.g., TBD-000000027 is the CAS number used for the class PCBs) (Supplement One).^16^

These compounds/metals may be present in high concentrations in the soil or ground water at Superfund sites throughout the U.S.^16^ The location of many of these Superfund sites are in regions of the U.S. where there is a continuing rise in temperature and where there are various social factors of concern.^20^

Cancer potential of individual compounds and metals was based on the NTP RoC and IARC data. The cancer potential of the 30 most frequent compounds and metals found at U.S. Superfund sites were assessed based on evaluations from the NTP RoC and IARC (Table 1). The number of sites where the 30 most frequently found compounds or metals are found is summarized in Figures 1 and 2.

**Figure 1:**
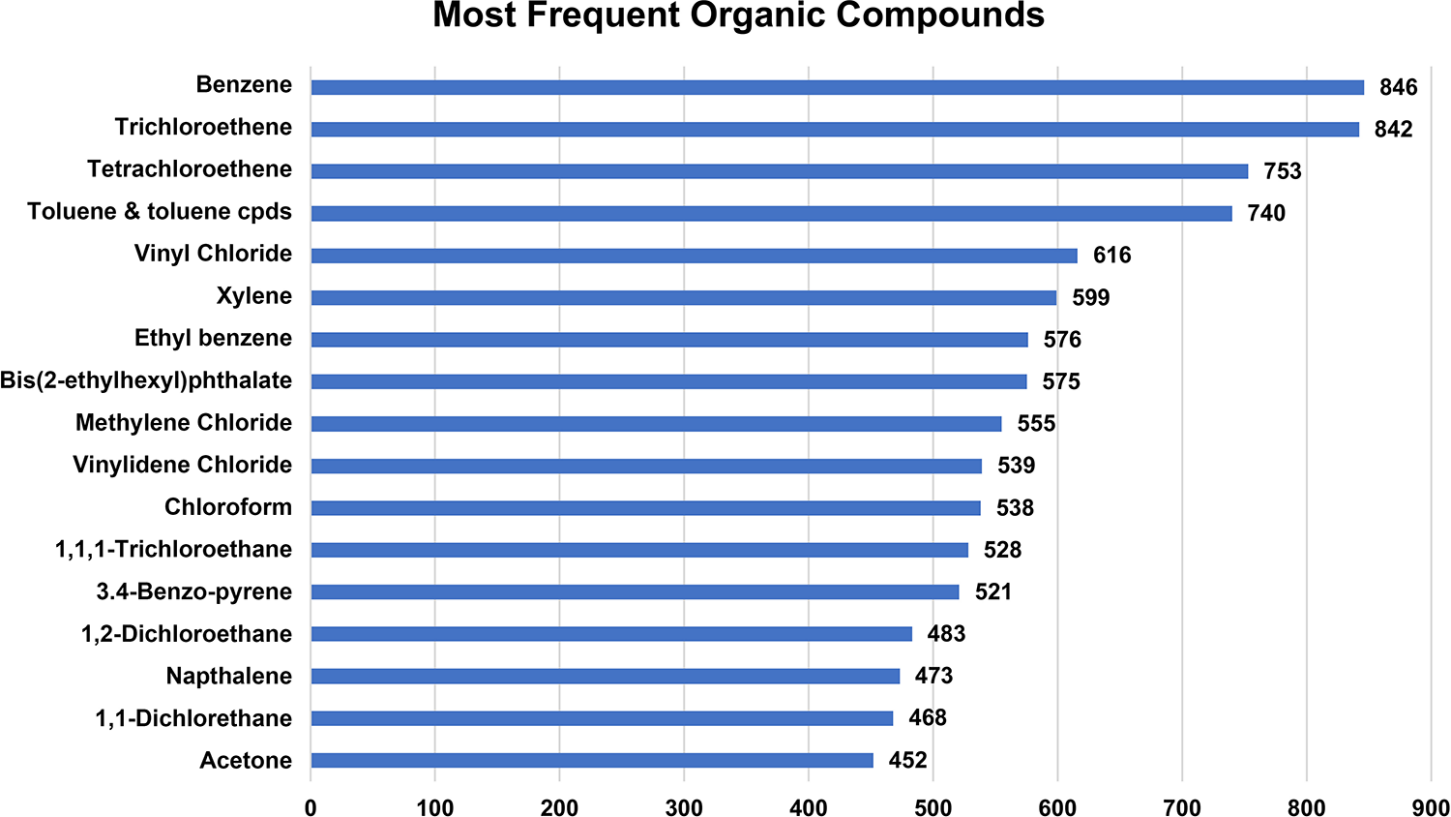
Most frequently found organic compounds at 1582 U.S. Superfund sites.

**Figure 2:**
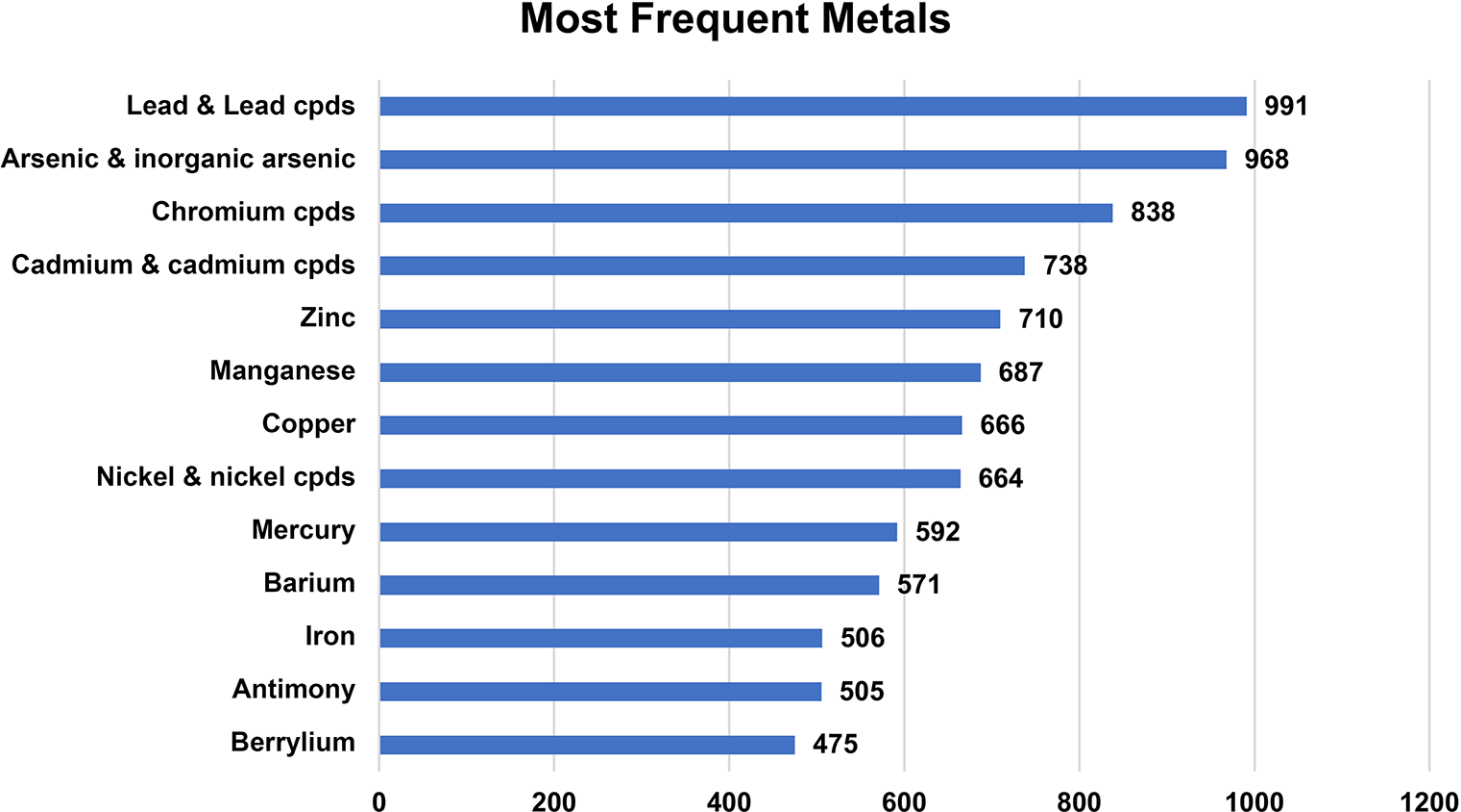
Most frequently found metals at 1582 U.S. Superfund sites.

**Table 1:** Cancer Classification of Single Compounds and Metals Frequently Found at U.S. Superfund Sites

Cas. No.	Compound/Metal	Number of U.S. Superfund Sites^a^	NTP Report on Carcinogens (RoC) Cancer Classification^b^	IARC Cancer Classification^c^	NTP *Salmonella* Results^d^	NTP Technical Report Number^e^	NTP Male Rat Cancer Target Organs^e^	NTP Female Rat Cancer Target Organs^e^	NTP Male Mouse Cancer Target Organs^e^	NTP Female Mouse Cancer Target Organs^e^
7439-92-1	Lead Compounds	991	Reasonably anticipated – humans – lung, stomach, and urinary-bladder cancer	Group 2A	Not evaluated	Not tested in NTP cancer studies	Not evaluated	Not evaluated	Not evaluated	Not evaluated
				Benign and malignant kidney tumors (adenoma, carcinoma, and adenocarcinoma) were most frequently associated with lead exposure, and tumors of the brain, hematopoietic system, and lung were reported in some studies (IARC 1980, 1987).						
7440-38-2	Arsenic and Inorganic Arsenic	968	Known – humans skin, lung, digestive tract, liver, urinary bladder, kidney, and lymphatic and hematopoietic systems	Group 1	Not evaluated	NTP Research Studies^21^	+	+	+	+
				Lung, urinary bladder, skin			Enhances carcinogenicity of known carcinogens	Enhances carcinogenicity of known carcinogens	Liver, Adrenal, Lung	Liver, Adrenal, Lung, Ovary
71-43-2	Benzene	846	Known – humans (leukemia)	Group 1	Negative	TR-289	+	+	+	+
			Animals – multiple organs	Leukemia, Lymphoma			Oral Cavity, Skin, Zymbal’s Gland	Oral Cavity, Zymbal’s Gland	Harderian Gland, Hematopoietic System, Lung, Preputial Gland, Zymbal’s Gland	Hematopoietic System, Lung, Mammary Gland, Ovary Zymbal’s Gland
79-01-6	Trichloroethene (Trichloroethylene)	842	Known – humans, kidney	Group 1	Negative	TR-002	–	–	+	+
			Animals – kidney and other organs	Kidney					Liver	Liver
	Trichloroethene				Not evaluated	TR 243	+	–	+	+
							Kidney, tubular cell		Liver	Liver
	Trichloroethene				Not evaluated	TR 273 (inadequate study)	Not evaluated	Not evaluated	Not evaluated	Not evaluated
7789-12-0	Chromium compounds	838	Known – human (lung)	Group 1	Positive	TR 546 (chromium VI)	+	+	+	+
	Chromium VI	128	Animals (lung) (chromium VI)	Lung (chromium VI) 18540-29-9			Oral cavity – squamous cell neoplasms	Oral cavity – squamous cell neoplasms	Small intestine neoplasms	Small intestine neoplasms
127-18-4	Tetrachloroethene (Tetrachloroethylene)	753	Reasonably anticipated	Group 2A	Negative	TR-013	Inadequate	Inadequate	+	+
			Animals – sufficient evidence (multiple organs)	Urinary Bladder					Liver	Liver
	Tetrachloroethene (Tetrachloroethylene)				Negative	TR 311	+	+	+	+
							Mononuclear cell leukemia, Renal tubular cell neoplasms	Mononuclear cell leukemia	Liver	Liver
95-80-7	2,4-Diaminotoluene	740 (other forms of toluene)	Reasonably anticipated	Group 2B	Positive	TR 162	+	+	–	+
			Animals sufficient evidence (multiple organs)				Liver	Liver, Mammary gland		Liver
26471-62-5	Toluene Diisocyanates	740	Reasonably anticipated	Group 3	Positive	TR 251	+	+	–	+
			Animals sufficient evidence (multiple organs)				Subcutaneous fibromas and fibrosarcomas (combined) pancreatic acinar cell adenomas	Subcutaneous fibromas and fibrosarcomas (combined) pancreatic islet cell adenomas, neoplastic nodules of the liver, and mammary gland fibroadenomas		Hemangiomas or hemangiosarcomas (combined) as well as hepatocellular adenomas.
7440-43-9	Cadmium and Cadmium Compounds	738	Known – human (lung)	Group 1	Not evaluated	Not tested by NTP in cancer studies	Not evaluated	Not evaluated	Not evaluated	Not evaluated
			Sufficient – animals (lung & other organs)	Lung						
7440-66-6	Zinc (Dietary Zinc)	710	Not evaluated	Not evaluated	Not evaluated	TR 592	–/+	–	–	–
7439-96-5	Manganese	687	Not evaluated	Not evaluated	Not evaluated	Not tested in NTP cancer studies	Not evaluated	Not evaluated	Not evaluated	Not evaluated
10034-96-5	Manganese(II) Sulfate Monohydrate		Not evaluated	Not evaluated		TR 428	–	–	–/+	–/+
7440-50-8	Copper	666	Not evaluated	Not evaluated	Not evaluated	Not tested in NTP cancer studies	Not evaluated	Not evaluated	Not evaluated	Not evaluated
7440-02-0	Nickel and Nickel Compounds	664	Known	Lung, Nasal Cavity	Not evaluated					
			Lung							
10101-97-0	Nickel Sulfate Hexahydrate		Evaluated in nickel group		Negative	TR-454	–	–	–	–
12035-72-	Nickel Subsulfide		Evaluated in nickel group		Negative	TR-453	+	+	–	–
							Lung, Adrenal medulla	Lung, Adrenal medulla		
1313-99-1)	Nickel Oxide		Evaluated in nickel group		Negative	TR-451	+	+	–	–/+
							Lung, Adrenal medulla	Lung, Adrenal medulla		
75-01-4	Vinyl Chloride	616	Known	Group 1	Not evaluated	NTP Research Study^22^	Not evaluated	Hemangiosarcomas, hepatocellular carcinomas, and mammary gland carcinomas	ND	Hemangiosarcomas and mammary gland carcinomas, lung
			Liver	Liver and bile duct						
1330-20-7	Xylene	599	Not evaluated	Group 3	Negative	TR-327	–	–	–	–
7439-97-6	Mercury	592	Not evaluated	Group 3	Not evaluated	Not tested in NTP cancer studies	Not evaluated	Not evaluated	Not evaluated	Not evaluated
100-41-4	Ethylbenzene	576	Not evaluated	Group 2B	Negative	TR-466	+	+		
							Kidney, Tubular Cell, Testes	Kidney, Tubular Cell		
117-81-7	Bis(2-ethylhexyl) Phthalate	575	Reasonably anticipated	Group 2B	Negative	TR-601	Not evaluated	+	+	+
	(Di(2-ethylhexyl) Phthalate)							Liver	Liver	Liver
7440-39-3	Barium	571	Not evaluated	Not evaluated	Not evaluated	Not tested in NTP cancer studies	Not evaluated	Not evaluated	Not evaluated	Not evaluated
75-09-2	Methylene Chloride (Dichloromethane)	555	Reasonably anticipate (insufficient evidence in humans)	Group 2A	Positive	TR-306	+	+	+	+
							Mammary Gland	Mammary Gland	Lung, Liver	Lung, Liver
75-35-4	Vinylidene Chloride (1,1-Dichloroethene)	539	Reasonably anticipated	Group 2B	Negative	TR-582	+	+	+	+
							Kidney, Nasal Cavity, Malignant mesothelioma	Thyroid Gland Cell	Kidney	Hemangioma/Hemangioma sarcoma, Liver
								Mononuclear cell leukemia		
67-66-3	Chloroform	538	Reasonably anticipated	Group 2B	Negative	TR-000 (67-66-3)	+	–	+	–
							Kidney, Tubular Cell	(uncertain findings)	Liver	Liver
71-55-6	1,1,1-Trichloroethane	528	Reasonably Anticipated	Group 3	Negative	TR-003	Not evaluated	Not evaluated	Not evaluated	Not evaluated
50-32-8	3,4-Benzo-pyrene	521	Included in Polyaromatic class as Reasonably anticipated based on animal data	Group 1	Positive	Not tested in NTP cancer studies	Not evaluated	Not evaluated	Not evaluated	Not evaluated
7439-89-6	Iron	506	Not evaluated	Not evaluated	Not evaluated	Not tested in NTP cancer studies	Not evaluated	Not evaluated	Not evaluated	Not evaluated
7440-36-0	Antimony	505	Reasonably anticipated	Group 2B	Negative	TR - 590	+	+	+	+
	Antimony Trioxide Form Examined by NTP 1309-64-4						Lung, Adrenal	Lung, Adrenal	Lung, Skin	Lung, Skin, Lymphoma
107-06-2	1,2-Dichloroethane	483	Reasonably anticipated	Group 2B	Positive	TR-055	+	+	Lung	+
							Forestomach, Subcutaneous Tissue, Vascular System	Mammary Gland		Lung, Mammary Gland, Uterus, cervix
7440-79-00	Beryllium	475	Known – lung cancer	Group 1	Negative	Not tested in NTP cancer studies	Not evaluated	Not evaluated	Not evaluated	Not evaluated
91-20-3	Naphthalene	473	Reasonably anticipated	Not evaluated	Negative	TR-500	+	+	Not evaluated	Evaluated
							Nasal cavity	Nasal cavity		
75-34-3	1,1-Dichloroethane	468	Reasonably anticipated	Not evaluated	Negative	TR-55	+	+	+	+
							Forestomach, hemangiosarcomas, skin	Mammary gland	Lung	Mammary gland uterus
67-64-3	Acetone	452	Not evaluated	Not evaluated	Not evaluated	Not evaluated	Not evaluated	Not evaluated	Not evaluated	Not evaluated

a U.S. Environmental Superfund information^23^ – total number of 1582 U.S. Superfund Sites included in this analysis.

b National Toxicology Program (NTP) Report on Carcinogens (RoC);^17^ RoC classifications: Known – known to be a human carcinogen; Reasonably anticipated – reasonably anticipated to be a human carcinogen.

c International Agency for Research on Cancer (IARC);^2^ IARC classification groups: Group 1 – Carcinogenic to humans; Group 2A – probably carcinogenic to humans; Group 2B – possibly carcinogenic to humans; Group 3 – nonclassifiable.

d NTP Genetic Toxicology *Salmonella* results.^18^

e NTP cancer study results: +, some or clear evidence of cancer; +/–, equivocal evidence of cancer; −, no evidence of cancer; NE, not evaluated.^6^

Of the 30 most frequently found compounds or metals, 8 were reported as known human carcinogens, 13 as reasonably anticipated to be human carcinogens, and 9 as not being evaluated by the NTP RoC (Table 1).

Of the 30 most frequently found compound or metal groups at U.S. Superfund sites, 8 were classified as known human carcinogens by the NTP RoC including: arsenic, benzene, trichloroethenes, chromium, beryllium, cadmium, nickels, and vinyl chloride.

Of the top 30 most frequently found groups of compounds or metals at U.S. Superfund sites, 13 were classified as reasonably anticipated to be human carcinogens by the NTP RoC including: lead, tetrachloroethene, toluene-based compounds, phthalate compounds, methylene chloride, vinylidene chloride, chloroform, 1,1,1-trichloroethane, benzopyrene, antimony, 1,2-dichloroethane, naphthalene, and dichloroethane.

Nine of the 30 most frequently found compounds or metals at the U.S. Superfund sites have not been evaluated by the NTP RoC including: zinc, manganese, copper, xylene, mercury, ethylbenzene, barium, iron, and acetone. Of these 9 compounds/metals not evaluated by the NTP RoC, one (ethylbenzene) has been evaluated by IARC and classified as group 2B (possibly carcinogenic to humans) (Figure 3).

**Figure 3:**
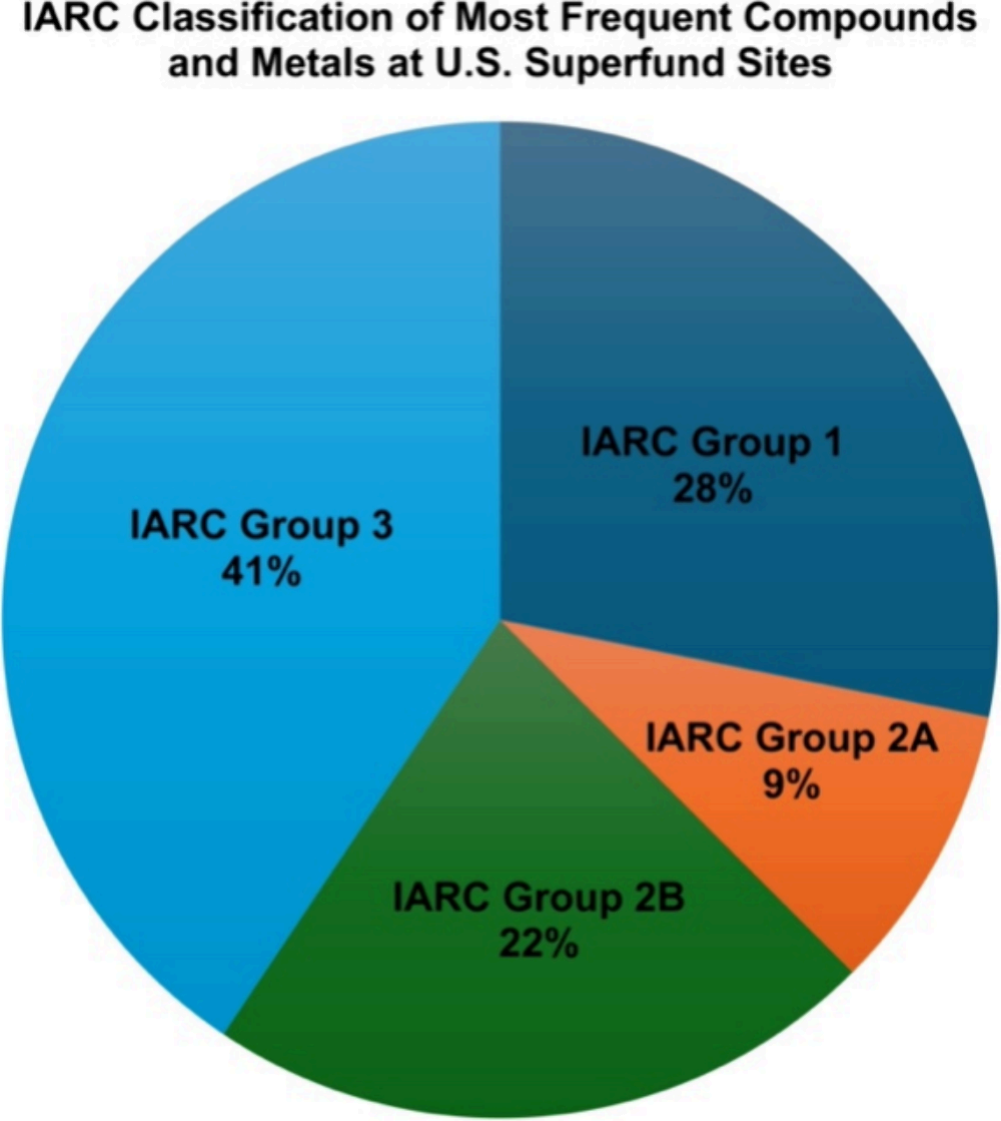
IARC classification of most frequently found compounds and chemicals at U.S. Superfund sites. IARC Group 1 = The agent is carcinogenic to humans; IARC Group 2A = The agent is probably carcinogenic to humans; IARC Group 2B = The agent is possibly carcinogenic to humans; and IARC Group 3 = The agent is not evaluated or classifiable as to its carcinogenicity to humans.

Cancer potential of compound and metal mixtures is based on the NTP RoC data. A total of 921 mixtures of three compounds/metals were identified at the 1582 U.S. Superfund sites (Supplement Two). The cancer potentials of the most frequently found compound/metal mixtures at U.S. Superfund sites are summarized based on evaluations from the NTP RoC (Table 2).

**Table 2:** Mixtures of Three Compound/Metals with Two or Three Known Human Carcinogens Frequently Found at U.S. Superfund Sites

No. of Superfund Sites	Mixtures of Three Compound/Metals
Mixtures of Three with Three Known Compound/Metal Human Carcinogens	
496	Nickel compounds, Cadmium, Arsenic compounds
451	Benzene, Arsenic compounds, Trichloroethene
433	Benzene, Cadmium, Arsenic compounds
424	Nickel compounds, Benzene, Arsenic compounds
420	Benzene, Vinyl Chloride, Trichloroethene
414	Cadmium, Arsenic compounds, Trichloroethene
391	Nickel compounds, Arsenic compounds, Trichloroethene
389	Nickel compounds, Benzene, Cadmium
386	Vinyl Chloride, Arsenic compounds, Trichloroethene
385	Beryllium, Cadmium, Arsenic compounds
379	Nickel compounds, Beryllium, Arsenic compounds
379	Benzene, Cadmium, Trichloroethene
368	Benzene, Vinyl Chloride, Arsenic compounds
366	Nickel compounds, Beryllium, Cadmium
366	Nickel compounds, Cadmium, Trichloroethene
360	Nickel compounds, Benzene, Trichloroethene
328	Benzene, Beryllium, Arsenic compounds
321	Cadmium, Vinyl Chloride, Arsenic compounds
Mixtures of Three with Two Known Compound/Metal Human Carcinogens	
611	Lead, Cadmium, Arsenic compounds
549	Nickel compounds, Lead, Arsenic compounds
518	Benzene, Lead, Arsenic compounds
517	Nickel compound, Lead, Cadmium
500	Tetrachloroethene, Benzene, Trichloroethene
478	Lead, Arsenic compounds, Trichloroethene
474	Tetrachloroethene, Arsenic compounds, Trichloroethene
461	Tetrachloroethene, Vinyl Chloride, Trichloroethene
455	Benzene, Lead, Cadmium
452	Benzene, Lead, Trichloroethene
433	Nickel compound, Benzene, Lead
433	Lead, Cadmium, Trichloroethene

There were 18 compound/metal mixtures for which all three mixture components are listed in the NTP RoC as known human carcinogens (Figure 4; Supplement Two).

**Figure 4:**
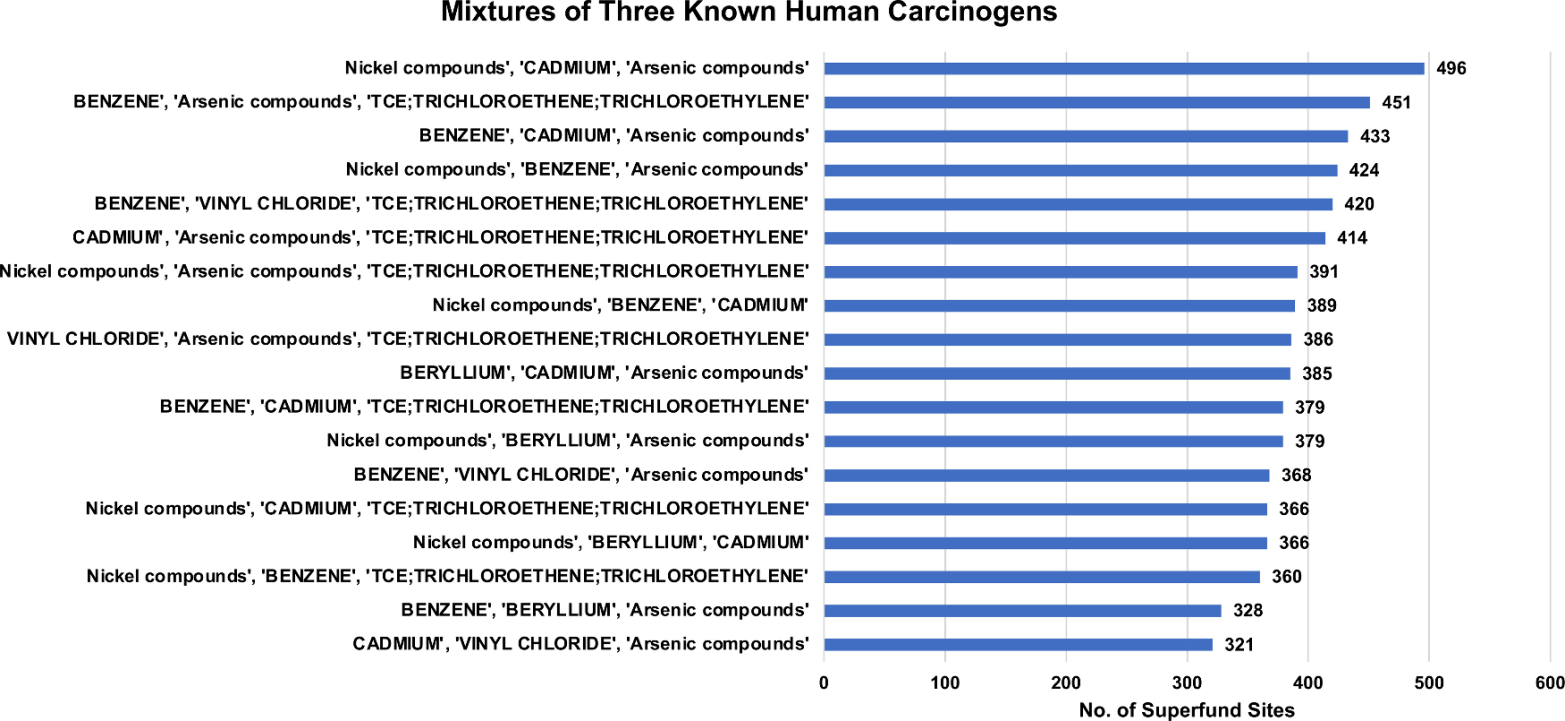
Most frequent mixtures of three known human carcinogens at U.S. Superfund sites.

There were 192 mixtures in which two of the three compound/metal components are listed in the NTP RoC as known human carcinogens (Supplement Two). The 18 mixtures with all components being human carcinogens by the NTP RoC and the 12 most frequent mixtures with two of the components being known human carcinogens are given in Table 2. The cancer target organ, as determined from experimental studies, varied depending on the compound/metal (Table 3), and cancer or disease could be caused at a variety of target sites including liver, lung, kidney, mammary gland, and other organs.

**Table 3:** Cancer Target Organs for Frequent U.S. Superfund Compound/Metal Mixtures

Mixture of Three	No. of Superfund Sites	Human Cancer Target Organ
Nickel, Cadmium, Arsenic	496	Nickel (lung)
		Cadmium (lung)
		Arsenic (skin, lung, digestive tract, liver, urinary bladder, kidney, and lymphatic and hematopoietic systems)
Benzene, Arsenic, Trichloroethene	451	Benzene (lymphoma, leukemia)
		Arsenic (skin, lung, digestive tract, liver, urinary bladder, kidney, and lymphatic and hematopoietic systems)
		Trichloroethene (kidney)
Benzene, Cadmium, Arsenic	433	Benzene (lymphoma, leukemia)
		Cadmium (lung)
		Arsenic (skin, lung, digestive tract, liver, urinary bladder, kidney, and lymphatic and hematopoietic systems)
Benzene, Vinyl Chloride, Trichloroethene	420	Benzene (lymphoma, leukemia)
		Vinyl chloride (liver and bile duct)
		Trichloroethene (kidney)
Arsenic, Vinyl Chloride, Trichloroethene	386	Arsenic (skin, lung, digestive tract, liver, urinary bladder, kidney, and lymphatic and hematopoietic systems)
		Vinyl chloride (liver and bile duct)
		Trichloroethylene (kidney)

Some of the commonly found mixtures at U.S. Superfund sites were nickel, arsenic, and cadmium (496 sites); benzene, arsenic, trichloroethene (451 sites); benzene, vinyl chloride, trichloroethene (420 sites); and arsenic, vinyl chloride, trichloroethene (386 sites). Compounds and metals associated with the development of liver cancer at the U.S. Superfund sites included vinyl chloride and arsenic. Those associated with the development of lung cancer included many of the metals, such as cadmium, nickel, arsenic, and beryllium. Benzene is associated with the development of lymphoma/leukemia.^3^ Thus, mixture exposure could cause cancer at multiple target sites.

## Discussion and Conclusions

Cancer hazards of compounds, metals, and compound/metal mixtures frequently found at U.S. Superfund sites were summarized and correlated with their potential to cause cancer based on results of NTP rodent cancer studies, the RoC monographs, and data from IARC. The individual components of a mixture may have different organ target sites of cancer or activate different cancer pathways (e.g., genotoxic pathways, nongenotoxic pathways, oxidative damage, etc.). Many of the compounds and metals found at U.S. Superfund sites have been reported to cause cancer at some of the most frequent sites for cancer in humans including the lung, liver, colon and rectum, urinary bladder, reproductive organs, or the lymphatic system.^3,24^ Thus, mixture exposure has the potential to cause cancer at multiple target organ sites.

The carcinogenic properties or preferential site of cancer induction by organic compounds is varied and is often dependent on the route, duration, and amount of exposure, metabolic processes, genotoxic or epigenetic factors, hormonal disruption, population susceptibility, and other biological parameters such as the target cell, DNA repair mechanisms, and tissue/tumor microenvironment.^25^ The existence of compounds or metals may be complex in themselves within our 3-mixture paradigm, and although these compounds or metals may be commonly found together at U.S. Superfund sites, this does not automatically imply equal coexposures; many other factors as outlined above must be factored into the exposures and cancer outcomes.

For example, halogenated chemicals are common chemicals found at U.S. Superfund sites, and many of these halogenated chemicals (e.g., trichloroethene, tetrachloroethene, and vinyl chloride) are reported by IARC to cause liver cancer.^26^ However, other halogenated chemicals may cause cancer at other target organ sites (e.g. trichloroethene is associated with kidney cancers^26^). There are different forms of metals at U.S. Superfund sites, and not all metal forms may have enough information for an IARC evaluation of their carcinogenic potential. For example, chromium VI is classified in IARC group 1 (carcinogenic to humans), while there is not enough information for an IARC classification of metallic chromium.^27^ Other metals at U.S. Superfund sites also have not been evaluated by IARC for carcinogenic potential (e.g., copper, xylene, mercury, barium, iron) because of a lack of sufficient toxicity studies.^28^

In general, the toxicity of mixtures has not been well studied especially the effects of long-term mixture exposures and cancer,^29^ and IARC generally reviews carcinogenic potential one compound at a time.^28^ It is recommended that, in order to adequately evaluate the effects of chemical mixtures, an integrated, systematic, and collaborative approach is needed across different disciplines, such as toxicology, epidemiology, exposure science, risk assessment, and statistics to make proper and accurate assessments of mixture exposures and outcomes.^29^ It is evident that humans experience complex exposures throughout their lifetime, and many of the current approaches for understanding the mechanisms of chemically induced carcinogenesis have not taken this into account. There are many challenges in designing experimental research studies to evaluate the effects of mixtures and understanding the possible outcomes to inform hazard or risk assessments; however, new concepts and approaches consisting of chemical screening, transgenic model-based, and disease-centered methods have been proposed for evaluating mixtures and cancer development.^30^

In this summary, some of the most frequent organic compounds found at Superfund sites that are classified as known human carcinogens include benzene, benzo­[a]­pyrene, bis­(2-ethylhexyl) phthalate, chloroform, 1,1-dichloroethene, 1,2-dichloroethane, ethylbenzene, methylene chloride, 1,1,1-trichloroethane, tetrachloroethene, trichloroethene, and vinyl chloride. The most frequent metals identified at Superfund sites are classified as known or reasonably anticipated to be human carcinogens and included arsenic, beryllium, cadmium, nickel, and lead.

Ten “key characteristics”^31^ are used to describe different mechanisms of carcinogenicity, and any one compound or metal may have several “key characteristics”. For example, benzene is thought to cause carcinogenic activity by a variety of mechanisms including immunosuppression, DNA mutations, metabolic alterations, and/or oxidative damage.^32^ Metals may cause oxidative damage leading to cancer.^33^ Compound and metal exposures acting by different cancer mechanisms may work together to exert a greater toxic and carcinogenic effect than exposure to one compound alone.

Cancer hazard evaluations have not been performed on compound/metal mixtures found at U.S. Superfund sites. The location of many of the U.S. Superfund sites are in regions in the U.S. where a continuing rise in temperature occurs and other social factors are present that may make populations particularly sensitive to cancer development.^20,34^

Toxicology and cancer studies of mixtures found at U.S. Superfund sites would provide needed information for cancer hazard evaluation. Such information could be used to develop strategies to protect the public, particularly segments of the population that live near U.S. Superfund sites. Mixture toxicology studies could include both *in vivo* and *in vitro* studies to provide data for identification of early cancer disease biomarkers of exposure.^35^ Use of these early cancer biomarkers would help in detecting cancer in vulnerable populations living near U.S. Superfund sites.

## Supplementary Material





## Notes

List and occurrence of individual compounds/metals at U.S. Superfund sites (XLSX)
List and Occurrence of Mixtures at U.S. Superfund Sites (XLSX)

## References

[1] Goodson W. H., Lowe L., Carpenter D. O., Gilbertson M., Manaf Ali A., Lopez de Cerain Salsamendi A., Lasfar A., Carnero A., Azqueta A., Amedei A. (2015). Assessing the carcinogenic potential of low-dose exposures to chemical
mixtures in the environment: the challenge ahead. Carcinogenesis.

[2] International Agency for Research on Cancer . IARC Cancer Classifications, 2024; https://monographs.iarc.who.int/cards_page/preamble-monographs/; https://monographs.iarc.who.int/agents-classified-by-the-iarc/.

[3] Bassan A., Alves V. M., Amberg A., Anger L. T., Beilke L., Bender A., Bernal A., Cronin M. T. D., Hsieh J. H., Johnson C. (2021). In silico approaches
in organ toxicity hazard assessment:
Current status and future needs for predicting heart, kidney and lung
toxicities. Comput. Toxicol.

[4] Center for Disease Control . Leading causes of Death - United States; 2022; https://www.cdc.gov/nchs/fastats/leading-causes-of-death.htm.

[5] Nagisetty R. M., Autenrieth D. A., Storey S. R., Macgregor W. B., Brooks L. C. (2020). Environmental health
perceptions in a superfund community. J. Environ.
Manage.

[6] National Toxicology Program. NTP Technical Reports; 2024; https://ntp.niehs.nih.gov/publications/reports/tr?type=Technical%20Report.

[7] National Toxicology Program. 15th Report of Carcinogens; 2024; https://ntp.niehs.nih.gov/whatwestudy/assessments/cancer/roc/index.html?utm_source=direct&utm_medium=prod&utm_campaign=ntpgolinks&utm_term=roc15. 42475452

[8] U.S. Environmental Protection Agency . What is Superfund? https://www.epa.gov/superfund/what-superfund 2020 (accessed).

[9] U.S. Congress . Comprehensive Environmental Response, Compensation, and Liability Act; 1980; https://www.govinfo.gov/content/pkg/USCODE-2011-title42/html/USCODE-2011-title42-chap103.htm. 10132830

[10] Burwell-Naney K., Zhang H., Samantapudi A., Jiang C., Dalemarre L., Rice L., Williams E., Wilson S. (2013). Spatial disparity in
the distribution of superfund sites in South Carolina: an ecological
study. Environ. Health.

[11] Presidential Documents. Executive order 12898 - Federal actions to address environmental justice in minority populations and low-income populations. Federal Register 1994, 59, No. 32.

[12] Burda M., Harding M. (2014). Environmental Justice: Evidence from
Superfund cleanupdurations. Journal of Economic
Behavior & Organization.

[13] Bopp S. K., Kienzler A., Richarz A. N., van der
Linden S. C., Paini A., Parissis N., Worth A. P. (2019). Regulatory
assessment and risk management of chemical mixtures: challenges and
ways forward. Crit Rev. Toxicol.

[14] Harris J. R., Hjelmborg J., Adami H. O., Czene K., Mucci L., Kaprio J. (2019). The Nordic Twin Study on Cancer -
NorTwinCan.. Twin Res. Hum Genet.

[15] National Cancer Institute . Environmental Carcinogens and Cancer Risk; 2024; https://www.cancer.gov/about-cancer/causes-prevention/risk/substances/carcinogens.

[16] U.S. Environmental Protection Agency . List 10 - Contaminants at CERCLIS Sites; 2024; https://www.epa.gov/superfund/list-10-contaminants-cerclis-sites.

[17] National Toxicology Program. Report of Carcinogens; 2024; https://ntp.niehs.nih.gov/whatwestudy/assessments/cancer/roc/index.html?utm_source=direct&utm_medium=prod&utm_campaign=ntpgolinks&utm_term=roc15.

[18] National Toxicology Program. Genetic Toxicology; 2024; https://ntp.niehs.nih.gov/whatwestudy/testpgm/genetic/index.html.

[19] Ashby J., Tennant R. W. (1991). Definitive relationships
among chemical structure,
carcinogenicity and mutagenicity for 301 chemicals tested by the U.S.
NTP. Mutat Res..

[20] National Centers for Environmental Information . Climate Monitoring; 2022; https://www.ncdc.noaa.gov/climate-monitoring/#temp.

[21] Tokar E. J., Benbrahim-Tallaa L., Ward J. M., Lunn R., Sams R. L., Waalkes M. P. (2010). Cancer in experimental animals exposed
to arsenic and arsenic compounds. Crit Rev.
Toxicol.

[22] Drew R. T., Boorman G. A., Haseman J. K., McConnell E. E., Busey W. M., Moore J. A. (1983). The effect of age and exposure duration
on cancer induction by a known carcinogen in rats, mice, and hamsters. Toxicol Appl. Pharmacol.

[23] U.S. Environmental Protection Agency . Contaminants at CERCLIS Sites (List 10), Version 1.02; 2021; https://www.epa.gov/superfund/list-10-contaminants-cerclis-sites (accessed 2021).

[24] Siegel R.
L., Miller K. D., Fuchs H. E., Jemal A. (2021). Cancer Statistics,
2021. CA Cancer J. Clin.

[25] Stewart, B. A. Mechanisms of carcinogenesis: from initiation and promotion to the hallmarks. In Tumor Site Concordance and Mechanisms of Carcinogenesis; Baan, R. A. , Stewart, B. A. , Straif, K. , Eds.; International Society Research on Cancer, 2019; Vol. 165, pp 93–106. 33979080

[26] International Agency for Research on Cancer . List of classifications by cancer sites with sufficient or limited evidence in humans; IARC Monographs Volumes 1–137; 2025; https://monographs.iarc.who.int/wp-content/uploads/2019/07/Classifications_by_cancer_site.pdf.

[27] International Agency for Research on Cancer . Arsenic, metals, fibres, and dusts; 2012; volume 100C, https://publications.iarc.fr/Book-And-Report-Series/Iarc-Monographs-On-The-Identification-Of-Carcinogenic-Hazards-To-Humans/Arsenic-Metals-Fibres-And-Dusts-2012.

[28] International Agency for Research on Cancer . Agents Classified by the IARC Monographs, Volumes 1–137; 2025; https://monographs.iarc.who.int/agents-classified-by-the-iarc/.

[29] Hernández A. F., Tsatsakis A. M. (2017). Human exposure
to chemical mixtures: Challenges for
the integration of toxicology with epidemiology data in risk assessment. Food Chem. Toxicol.

[30] Rider C. V., McHale C. M., Webster T. F., Lowe L., Goodson W. H., La Merrill M. A., Rice G., Zeise L., Zhang L., Smith M. T. (2021). Using the Key Characteristics of
Carcinogens to Develop
Research on Chemical Mixtures and Cancer. Environ.
Health Perspect..

[31] Smith M. T., Guyton K. Z., Gibbons C. F., Fritz J. M., Portier C. J., Rusyn I., DeMarini D. M., Caldwell J. C., Kavlock R. J., Lambert P. F. (2016). Key Characteristics of Carcinogens as a Basis
for Organizing Data on Mechanisms of Carcinogenesis. Environ. Health Perspect.

[32] Costa-Amaral I. C., Carvalho L. V. B., Santos M. V. C., Valente D., Pereira A. C., Figueiredo V. O., Souza J. M., Castro V. S., Trancoso M. F., Fonseca A. S. A. (2019). Environmental
Assessment and Evaluation of Oxidative
Stress and Genotoxicity Biomarkers Related to Chronic Occupational
Exposure to Benzene. Int. J. Environ. Res. Public
Health.

[33] Ercal N., Gurer-Orhan H., Aykin-Burns N. (2001). Toxic metals and oxidative stress
part I: mechanisms involved in metal-induced oxidative damage. Curr. Top Med. Chem..

[34] Gordon C. J., Johnstone A. F., Aydin C. (2014). Thermal stress and toxicity. Compr Physiol.

[35] United Nations Environment Programme. Knowledge management and information sharing for the sound management of industrial chemicals; 2021;http://www.saicm.org/Portals/12/Documents/EPI/Knowledge_Information_Sharing_Study_UNEP_ICCA.pdf.

